# Early onset of efficacy with fremanezumab in patients with episodic and chronic migraine: subanalysis of two phase 2b/3 trials in Japanese and Korean patients

**DOI:** 10.1186/s10194-022-01393-0

**Published:** 2022-02-09

**Authors:** Takao Takeshima, Masami Nakai, Yoshiyuki Shibasaki, Miki Ishida, Byung-Kun Kim, Xiaoping Ning, Nobuyuki Koga

**Affiliations:** 1 Headache Center, Department of Neurology, Tominaga Hospital, 1-4-48 Minatomachi Naniwa-ku, Osaka-shi, Osaka, 556-0017 Japan; 2 Medical Affairs, Otsuka Pharmaceutical Co., Ltd., 3-2-27 Otedori, Chuo-ku, Osaka, 540-0021 Japan; 3 Medical Affairs, Otsuka Pharmaceutical Co., Ltd., Shinagawa Grand Central Tower, 2-16-4 Konan, Minato-ku, Tokyo, 108-8242 Japan; 4 Headquarters of Clinical Development, Otsuka Pharmaceutical Co., Ltd., 3-2-27 Otedori, Chuo-ku, Osaka, 540-0021 Japan; 5 Nowon Eulji Medical Center, Eulji University School of Medicine, 68 Hangeulbiseok-ro, Nowon-gu, Seoul, 01830 Republic of Korea; 6 Speciality Clinical Development, Teva Branded Pharmaceutical Products R&D, Inc., 145 Brandywine Pkwy, West Chester, PA 19380 USA; 7 Medical Affairs, Otsuka Pharmaceutical Co., Ltd., 463-10 Kagasuno, Kawauchi-cho, Tokushima, 771-0192 Japan

**Keywords:** Calcitonin gene-related peptide, Chronic migraine, Episodic migraine, Fremanezumab, Japanese, Korean, Early onset

## Abstract

**Background:**

Early onset of action has become recognized as an important efficacy feature of preventive migraine treatment, which can help overcome adherence issues commonly associated with older medications. Preventive treatments that target the calcitonin gene-related peptide (CGRP) or the CGRP receptor have been previously shown to provide early onset of action.

**Methods:**

This subanalysis of primary endpoints of two separate phase 2b/3 studies sought to determine the onset of action of fremanezumab in Japanese and Korean patients with episodic migraine (EM) and chronic migraine (CM).

**Results:**

In EM patients (*n* = 357), both fremanezumab quarterly and fremanezumab monthly led to greater reductions in weekly migraine days (days/week) than placebo from the first week after the initial injection and thereafter during the remainder of the study period. Similarly, CM patients (*n* = 571) had a greater reduction in headache days of at least moderate severity (days/week) with fremanezumab (total) than placebo. The percentage of patients with a migraine day (EM) or headache day at least moderate severity (CM) was lower in those treated with fremanezumab than placebo and this effect was apparent from as early as Day 2 (1 day after first injection).

**Conclusions:**

These results suggest that fremanezumab has an early onset of action, as noted in previous post hoc analyses of anti-CGRP monoclonal antibodies.

**Trial registration:**

ClinicalTrials.gov. NCT03303092, Registered 5 October 2017, NCT03303079, Registered 5 October 2017.

## Introduction

Both chronic migraine (CM) and episodic migraine (EM) are associated with significant pain and disability as well as impairments in quality of life, functioning, and interpersonal relationships [1–6]. Existing oral preventive treatments are limited by suboptimal efficacy, adverse events, and poor adherence [7–12]. Issues with adherence relate to both tolerability and poor efficacy, which in turn is associated with delays in gaining maximum effect during dose titration [13, 14]. One result of this is a high rate of treatment discontinuation [14]. Therefore, to overcome adherence problems, there is a strong need for preventive treatment that is both well tolerated and has an early onset of action [15].

More recently, preventive treatments that target the calcitonin gene-related peptide (CGRP) or the CGRP receptor have been extensively investigated. Among these, monoclonal antibodies that target the CGRP pathway have the advantage of being highly specific in their mechanism of action [14]. The rapidity of onset of monoclonal antibodies against CGRP has been the focus of several studies. In the earliest of these studies, post-hoc analyses of two pivotal trials of erenumab for CM (*n* = 667) and EM (*n* = 955) were conducted [16]. Rapid onset of efficacy of erenumab compared with placebo was observed in terms of change from baseline in weekly migraine days (WMD) and achievement of ≥50% reduction in WMD. Furthermore, a fewer erenumab-treated patients experienced migraine on a daily basis compared with placebo during the first week of treatment. Analyses based on two double-blind, randomized, Phase 3 studies comparing galcanezumab with placebo in EM patients (EVOLVE-1, EVOLVE-2) found galcanezumab-treated patients had significantly higher odds of having fewer migraine headache days in the first week and in each subsequent week compared with placebo [17]. Onset of effect was seen as early as the first day after injection [17, 18]. A post-hoc analysis based on the CONQUER phase 3b study of patients with prior treatment failure found that, compared with placebo, galcanezumab-treated patients had a significantly greater reduction in monthly migraine headache days starting at month 1, and in weekly migraine headache days starting at week 1 with the early onset of effect of galcanezumab considered to begin the day after treatment initiation [19]. Onset of efficacy for fremanezumab in CM was assessed as part of the phase 3 HALO trial [20]. During the 4-week period after the first dose, the mean number of monthly headache days of at least moderate severity was reduced for the all-fremanezumab group compared with the placebo group. Treatment effects were observed at week 1 for the all-fremanezumab group, with separation from placebo by Day 2 (1 day after first injection). Monthly average number of migraine days and the mean number of monthly headache hours showed similar effects.

Two recent phase 2b/3 studies have established the efficacy of fremanezumab in Japanese and Korean patients with EM or CM, respectively [21, 22]. Among patients with CM (*n* = 571), the least-squares mean (±standard error [SE]) reduction in the average number of headache days of at least moderate severity per month during 12 weeks (primary endpoint) was significantly greater with fremanezumab monthly (− 4.1 ± 0.4) and fremanezumab quarterly (− 4.1 ± 0.4) than with placebo (− 2.4 ± 0.4) [21]. Similarly, among patients with EM (*n* = 357), least-squares mean (±SE) reductions in the average number of migraine days per month during 12 weeks (primary endpoint) were significantly greater with fremanezumab monthly (− 4.0 ± 0.4, *n* = 121) and fremanezumab quarterly (− 4.0 ± 0.4, *n* = 117) than with placebo (− 1.0 ± 0.4, *n* = 116; *P* < 0.0001 for both comparisons) [22]. Improvements in secondary endpoints and a similar rate of adverse events, except injection-site reactions in the case of EM patients, with placebo were also observed with fremanezumab in both studies [21, 22].

Based on this background of previous studies, this subanalysis of two phase 2b/3 studies represents the first study to investigate the onset of efficacy of the CGRP monoclonal antibody, fremanezumab, in East Asian populations.

## Methods

### Study design

**Fig. 1:**
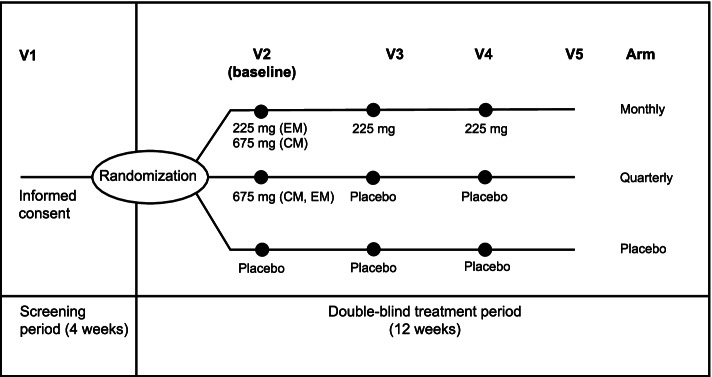
Study schema in CM and EM patients

This study represents a subanalysis of two multicenter, randomized, double-blind, placebo-controlled, parallel-group trials in Japanese and Korean patients with CM (Clinicaltrials.gov, NCT03303079) and EM (NCT03303092). The study design, populations, inclusion and exclusion criteria have been published in full previously. In brief, the EM trial randomly assigned 357 patients to subcutaneous fremanezumab monthly (225 mg at baseline, weeks 4 and 8), fremanezumab quarterly (675 mg at baseline and placebo at weeks 4 and 8), or matching placebo (Fig. 1). The primary endpoint of the EM trial was the mean change from baseline in the monthly average number of migraine days during the 12-week treatment period after the first dose. The CM trial randomized 571 patients to subcutaneous fremanezumab monthly (675 mg at baseline and 225 mg at weeks 4 and 8), fremanezumab quarterly (675 mg at baseline and placebo at weeks 4 and 8), or matching placebo (Fig. 1). The primary endpoint of the CM trial was the mean change from baseline in the monthly (28-day) average number of headache days of at least moderate severity during the 12 weeks after the first dose. These studies included prespecified secondary and exploratory analyses and post hoc analyses not previously specified for results up to 1 week.

### Study objective and outcomes

The overall objective of the subanalyses was to evaluate the efficacy of fremanezumab during the initial 4 weeks of treatment in patients with EM or CM.

In EM patients, the change in monthly migraine days (MMD, days/month) and the change in WMD (days/week) per week during the first 4 weeks from the first dose were assessed. In addition, EM patients were assessed in terms of the percentage of patients with a migraine day (from day 1 [day of the first injection] to day 7). EM patients were considered in terms of response to fremanezumab quarterly and fremanezumab monthly separately.

In CM patients, the change in headache days of at least moderate severity (days/month) and the change in headache days of at least moderate severity (days/week) per week during the first 4 weeks from the first dose were assessed. Patients with CM were also assessed in terms of the percentage of patients with a headache day of at least moderate severity (from day 1 to day 7). CM patients were considered in terms of response to fremanezumab quarterly and fremanezumab monthly combined.

### Statistics

An ANCOVA model was used to show the mean change in MMD (EM patients) and monthly headache days of at least moderate severity (CM patients) during the 4-week period from the first dose. An MMRM analysis was used to estimate the mean change from baseline in WMD (EM patients) and the change in weekly headache days of at least moderate severity (CM patients) per week during the 4-week period from the first dose. The percentages of patients with a migraine day (from day 1 to day 7) in EM patients and with a headache day of at least moderate severity (from day 1 to day 7) in CM patients were also calculated as part of post hoc analyses. Finally, two-sided 95% confidence intervals (CI) and *P* values with statistical significance set at *P* < 0.05 were constructed for the least-squares mean (LSM) and standard error of the mean (SEM) differences between each fremanezumab group and the placebo group. A headache day of at least moderate severity and a migraine day were normalized to 28 days for the monthly analysis and 7 days for the weekly analysis.

For CM patients, analyses of changes during the first 4 weeks after the first dose compared the placebo group with the “all-fremanezumab” group, which consisted of patients from both the fremanezumab quarterly and monthly groups. In both CM and EM patients, monthly variables with < 10 days of data and weekly variables with < 3 days of data were considered missing.

SAS version 9.4 (SAS Institute, Cary, NC) was used for all statistical calculations.

## Results

### Subject disposition and baseline characteristics

In total, 357 patients with EM and 571 patients with CM were included in the EM and CM phase 2b/3 studies. Patient baseline characteristics have been reported previously but are summarized in Table 1.

**Table 1:** Patient demographics and clinical characteristics

	**EM, Study NCT03303092**			**CM, Study NCT03303079**	
	**Placebo**	**Fremanezumab**		**Placebo**	**Fremanezumab**
	**(*n*** **= 117)**	**Monthly (*n*** **= 121)**	**Quarterly (*n*** **= 119)**	**(*n*** **= 191)**	**Total (*n*** **= 380)**
Age, years, mean (SD)	44.2 (10.7)	44.4 (9.5)	41.9 (10.1)	42.1 (10.2)	43.1 (10.2)
Body mass index, mean (SD)	22.8 (3.5)	23.0 (4.0)	22.5 (3.4)	22.8 (3.4)	22.9 (3.8)
Female sex, n (%)	100 (85.5)	101 (83.5)	101 (84.9)	163 (85.3)	328 (86.3)
Disease history					
Time since onset of migraine, years, mean (SD)	19.4 (13.3)	22.0 (12.9)	18.3 (11.4)	19.0 (11.2)	18.5 (12.3)
Use of preventive migraine medication at baseline, yes, n (%)	22 (18.8)	24 (19.8)	23 (19.3)	41 (21.5)	79 (20.8)
	***n*** **= 117**	***n*** **= 121**	***n*** **= 118**	***n*** **= 191**	***n*** **= 378**
Disease characteristics during 28-day pretreatment period					
Number of days with headache of any severity and duration, mean (SD)	11.1 (2.5)	11.0 (2.1)	11.0 (2.5)	21.2 (4.3)	21.4 (4.0)
Number of headache days of at least moderate severity, mean (SD)	8.0 (2.8)	7.6 (2.5)	7.5 (2.8)	13.5 (5.0)	13.3 (5.4)
Number of migraine days, mean (SD)	9.0 (2.8)	8.6 (2.5)	8.7 (2.5)	15.4 (5.0)	15.8 (5.2)
Use of any acute headache medications, yes, n (%)	117 (100.0)	120 (99.2)	117 (98.3)	191 (100.0)	375 (98.7)
Use of migraine-specific acute headache medications, yes, n (%)	114 (97.4)	115 (95.0)	110 (92.4)	177 (92.7)	352 (92.6)

*SD* Standard deviation

### Efficacy

**Fig. 2:**
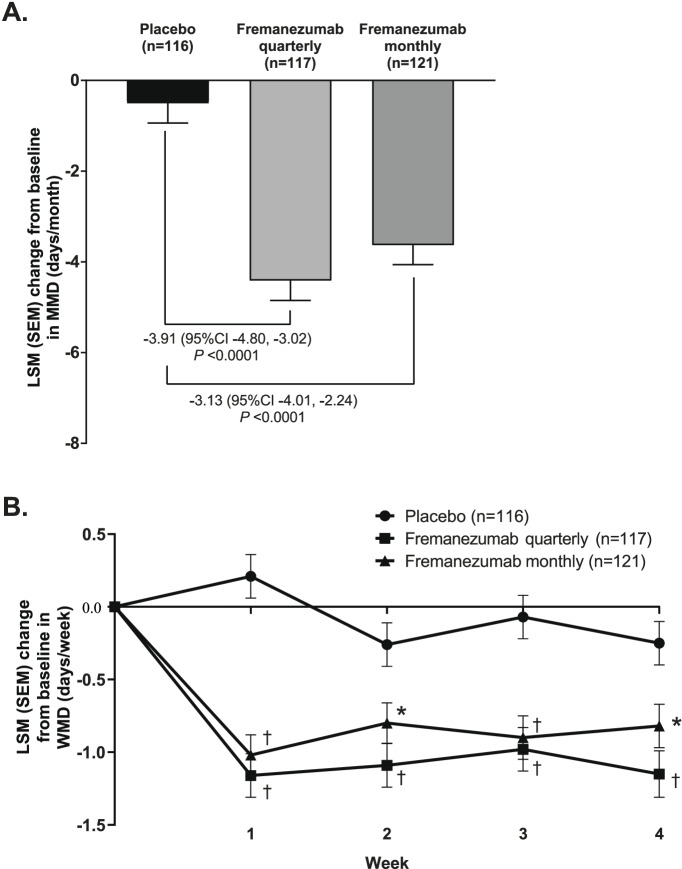
Change in (**A**) MMD and (**B**) WMD in EM patients. Change in MMD represents mean change from baseline during the 4-week period from the first dose (ANCOVA analysis) while change in WMD represents mean change per week during the 4-week period from the first dose (MMRM analysis). An asterisk denotes *P* < 0.05 and a dagger *P* < 0.0001 for the comparison with placebo. Abbreviations: ANCOVA, analysis of covariance; LSM, least-squares mean; MMD, monthly migraine days; MMRM, mixed-effects model for repeated measures; WMD, weekly migraine days

Regarding EM patients, both fremanezumab quarterly and fremanezumab monthly led to statistically significant greater reductions from baseline in MMD compared with placebo. Figure 2A shows the mean change in MMD by ANCOVA analysis during the 4-week period after the first dose, at which point the mean (SD) baseline was 9.0 (2.8), 8.6 (2.5) and 8.7 (2.5) days in the placebo, fremanezumab monthly, and fremanezumab quarterly groups, respectively. The LSM (SEM) change from baseline in MMD was equivalent to − 4.41 (0.44) days for fremanezumab quarterly, − 3.63 (0.43) days for fremanezumab monthly, and − 0.50 (0.44) days for placebo. This equated to a difference in LSM change from baseline of − 3.91 (95% CI −4.80, − 3.02; *P* < 0.0001) days/month for fremanezumab quarterly versus placebo and − 3.13 (95% CI −4.01, − 2.24; *P* < 0.0001) days/month for fremanezumab monthly versus placebo. Figure 2B shows the mean change in WMD by MMRM analysis per week during the 4-week period after the first dose, at which the mean (SD) baseline was 2.2 (0.7), 2.2 (0.6), and 2.2 (0.6) days in the placebo, fremanezumab monthly, and fremanezumab quarterly groups, respectively. These also showed statistically significant differences for both fremanezumab quarterly and fremanezumab monthly versus placebo.

**Fig. 3:**
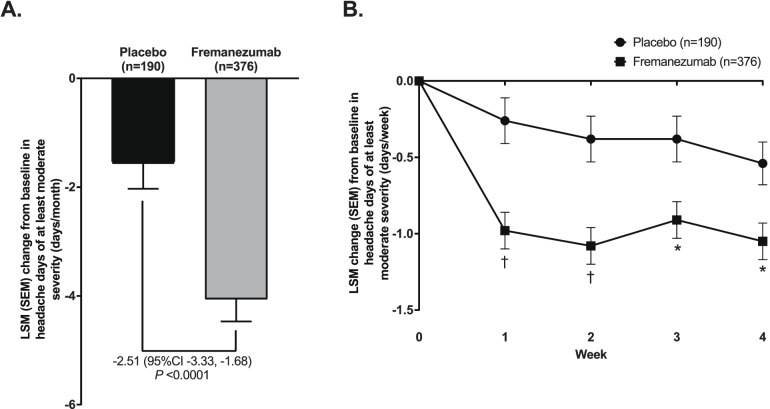
Change in (**A**) monthly and (**B**) weekly average headache days of at least moderate severity in CM patients. Change in monthly average number of headache days of at least moderate severity represents mean change from baseline during the 4-week period after the first dose (ANCOVA analysis) while change in the weekly average number of headache days of at least moderate severity represents mean change per week during the 4-week period from the first dose (MMRM analysis). An asterisk denotes *P* < 0.05 and a dagger *P* < 0.0001 for the comparison with placebo. Fremanezumab is the sum of fremanezumab monthly and fremanezumab quarterly groups. Abbreviations: ANCOVA, analysis of covariance; LSM, least-squares mean; MMRM, mixed-effects model for repeated measures

Similarly, in CM patients, fremanezumab led to a greater reduction from baseline in headache days of at least moderate severity (days/month) compared with placebo. Figure 3A shows the mean change in monthly headache days of at least moderate severity by ANCOVA analysis during the 4-week period from the first dose, at which the mean (SD) baseline was 13.5 (5.0), and 13.3 (5.4) days in the placebo and fremanezumab groups, respectively. Specifically, the fremanezumab group had an LSM (SEM) change from baseline of − 4.06 (0.41) days/month compared with − 1.56 (0.47) days/month for placebo. This equated to a difference in LSM change from baseline of − 2.51 (95%CI −3.33, − 1.68; *P* < 0.0001) days/month for fremanezumab versus placebo. Reductions in the number of headache days of at least moderate severity during the first 4 weeks showed statistically significant differences for fremanezumab versus placebo at each time point. Figure 3B shows the mean change in headache days of at least moderate severity by MMRM analysis per week during the 4-week period from the first dose, at which the mean (SD) baseline was 3.4 (1.2), and 3.3 (1.3) days in the placebo, and fremanezumab groups, respectively.

**Fig. 4:**
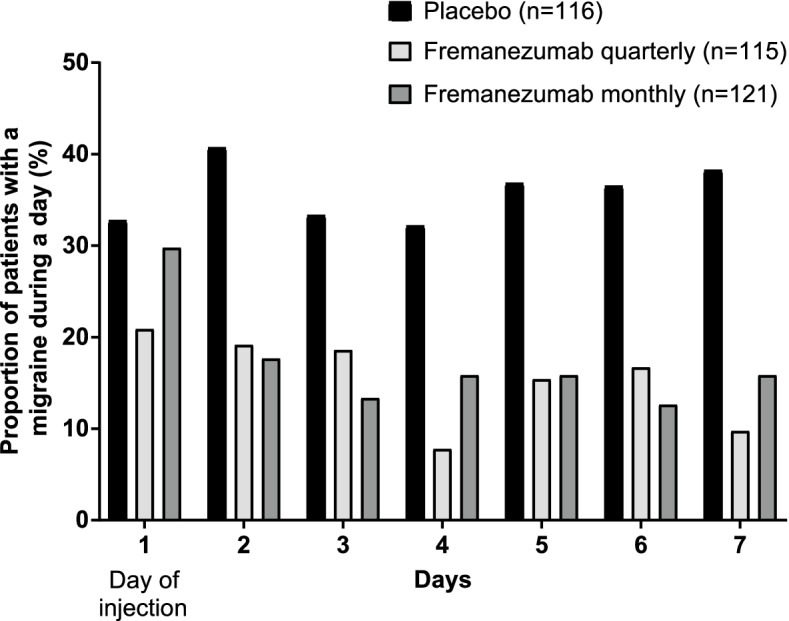
Percentage of EM patients reporting a migraine during a day from Day 1 to 7. Day 1 is the day of injection of study medications. *P* < 0.05 for difference with placebo from Day 1–7 for fremanezumab quarterly and from Day 2–7 for fremanezumab monthly

**Fig. 5:**
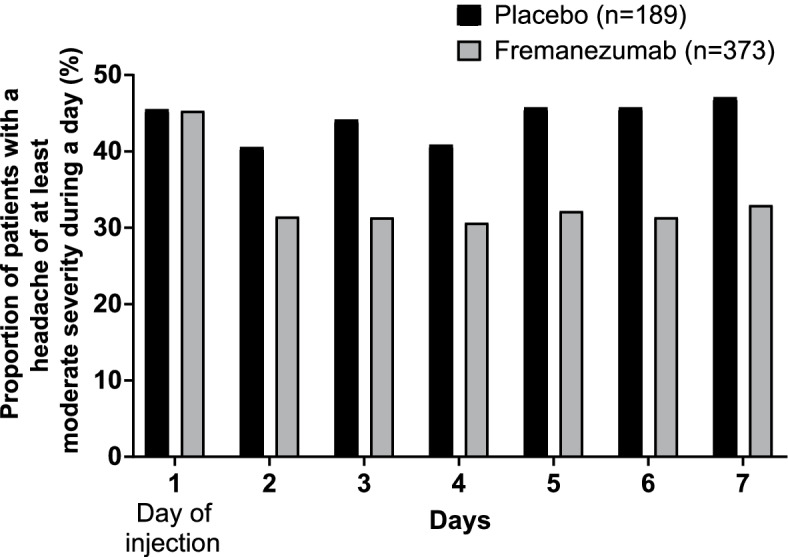
Percentage of CM patients reporting a headache during a day from Day 1 to 7. Headache in CM patients was defined as those of at least moderate severity. Day 1 is the day of injection of study medications. Fremanezumab is the sum of fremanezumab monthly and fremanezumab quarterly groups. *P* < 0.05 for difference with placebo from Day 2–7 for fremanezumab

Figure 4 and 5 show the percentage of patients with a migraine day and the percentage of patients with a headache day at least moderate severity from Day 1 (day of first injection) to Day 7 in EM patients and CM patients, respectively. In both populations, the percentage of patients was noticeably lower in fremanezumab-treated patients from as early as Day 2 (the day after the first injection) through to Day 7. In EM patients who received fremanezumab quarterly, the percentage of patients with a migraine day was lower as early as Day 1 (day of first injection).

## Discussion

Early onset of effect is rated as a highly important feature of migraine preventive treatment by patients and has been linked to improvements in adherence [23]. A previous post hoc analysis of the phase 3 HALO trial examining early onset found greater early reductions in the mean number of monthly headache days of at least moderate severity, monthly average number of migraine days and mean number of monthly headache hours for CM patients treated with fremanezumab versus placebo [20]. These subanalyses of early onset similarly found that preventive treatment with fremanezumab reduced MMD in EM patients and headache days of at least moderate severity in CM patients. Differences in efficacy endpoints between fremanezumab and placebo were sustained throughout the 4-week treatment period in these analyses. Further, the differential effect of fremanezumab versus placebo became evident during the first week of treatment and was even observed as early as the first day of injection in EM patients and the day after first injection in CM patients. These results suggest that fremanezumab has an early onset of action with clinical benefits for patients, with potential impact on patient satisfaction and adherence.

Two previous Phase 2b/3 studies in Japanese and Korean patients, which formed the basis of these subanalyses, established the efficacy of fremanezumab quarterly and monthly for periods up to 12 weeks [21, 22]. According to the primary endpoints, the reduction in the average number of migraine days (EM) or headache days of at least moderate severity (CM) per month during 12 weeks was significantly greater with fremanezumab monthly and fremanezumab quarterly than with placebo. Further, improvements in all secondary endpoints were also noted in both studies. Over long-term observation, both fremanezumab monthly and quarterly reduced the monthly number of migraine days in a 52-week, randomized, double-blind extension of the HALO trials [24]. A subanalysis of these long-term HALO results similarly found that fremanezumab monthly and quarterly led to sustained improvements in monthly migraine days and headache days of at least moderate severity throughout 12 months in Japanese patients with CM and EM.

Without minimizing the importance of long-term efficacy, there is also a clear patient preference for early efficacy benefits for migraine prevention [23], which has been a limitation of existing oral preventive medications. Indeed, in a patient survey, speed of onset was rated as the second most important aspect of preventive treatment besides actual effectiveness [23]. Switching between oral agents is a common practice to optimize therapy but persistence appears to actually worsen as patients cycle through agents [25].

Previously, early onset of efficacy has been shown with fremanezumab in CM patients [20], EM patients [26] and CM or EM patients with inadequate response to 2–4 prior migraine preventive treatment classes, including ≥1 inadequate response due to lack of efficacy [27, 28], erenumab in EM and CM patients [16] and galcanezumab in EM patients enrolled in the EVOLVE-1 and EVOLVE-2 studies [17], as well as in EM or CM patients with previous preventive medication failure [19]. Results from the present subgroup analysis were consistent with these previous studies of fremanezumab and other anti-CGRP pathway monoclonal antibodies. In the previous post hoc analysis during the first 4 weeks of a primary trial of fremanezumab in CM patients, the statistically significant difference in LSM (95% CI) change in headache days of at least moderate severity per month between fremanezumab total and placebo (− 2.3 [− 3.0, − 1.7], *P* < 0.0001) was highly similar to the difference in CM patients in this analysis (− 2.51 [− 3.33, − 1.68], *P* < 0.0001) [20]. In EM patients, corresponding differences in MMD between fremanezumab and placebo in this analysis were also significant for both fremanezumab monthly (− 3.13 [− 4.01, − 2.24], *P* < 0.0001) and fremanezumab quarterly (− 3.91 [− 4.80, − 3.02], *P* < 0.0001). Post hoc analyses of the FOCUS study in CM or EM patients with inadequate response to 2–4 prior migraine preventive treatment classes, including ≥1 inadequate response due to lack of efficacy also showed consistent results [27, 28]. In these analyses, the LSM (SE) change from baseline in the monthly average number of migraine days over the first 4 weeks of treatment was − 4.1 for both fremanezumab quarterly and fremanezumab monthly (versus − 0.6 for placebo, *P* < 0.0001 for both comparisons) [28]. Onset of action was also rapid with a significant reduction in the odds of experiencing a migraine with fremanezumab versus placebo noted from Day 2 (1 day after first injection) to Day 7 [27].

Significant differences between active treatment and placebo were also observed at 4 weeks in studies of other anti-CGRP pathway monoclonal antibodies [16, 17, 19]. Further analyses for each week up to 4 weeks found significant reductions in WMD or headache days of at least moderate severity per week in EM and CM patients, respectively, from week 1 in the present analysis, which continued through to week 4. Onset of effect at week 1 was also identified in the previous post hoc analysis of fremanezumab [20], as well as in studies of erenumab and galcanezumab [16, 17, 19]. Finally, additional analyses of efficacy within the first week were conducted in the present and previous analyses. In the present subanalysis, the percentage of patients with either a migraine day (EM patients) or headache day at least moderate severity (CM patients) was noticeably lower in fremanezumab-treated patients from as early as the day of the first injection in EM patients treated with fremanezumab quarterly or the day after the first injection in CM patients and EM patients treated with fremanezumab monthly. This, as previously noted, is consistent with the results for fremanezumab in post hoc analyses of the FOCUS study [27]. In the post hoc analysis of the phase 3b CONQUER study, onset of effect for galcanezumab was determined to occur the day following the first injection in EM or CM patients with previous preventive medication failures [19]. Similarly, in the analysis of the EVOLVE-1 and EVOLVE-2 studies in EM patients, the estimated proportion of patients experiencing migraine was significantly lower with galcanezumab compared with placebo from the day after the first injection [17]. Results for erenumab varied from day 3 to day 7 after injection depending on the dose and patient population [16].

Taken together, these results suggest that anti-CGRP pathway monoclonal antibodies, including fremanezumab, can provide early onset of action and thereby reduce the potential for patients to discontinue treatment. Discontinuation is a recognized disadvantage with oral preventive medications, which require daily adherence and titration to effect as well as potentially leading to headache chronification and medication overuse headaches through overuse of acute medications and tolerance [11, 14, 29]. Subanalyses of trials of anti-CGRP pathway monoclonal antibodies, including fremanezumab, have shown the potential of these agents to reduce the use of acute migraine treatment and lower the potential for medication overuse headaches [30, 31].

One of the main limitations of this subanalysis is that only Japanese and Korean patients were included. Nevertheless, the results are consistent with similar analysis from global study population. Further, this study is not powered to confirm treatment differences from placebo at early time points. Despite this limitation, substantial treatment differences were still demonstrated. Finally, assessment of migraine days and headache days of at least moderate severity were based on headache diary and therefore the influence of subjectivity in individual patient assessment cannot be ruled out.

In conclusion, fremanezumab shows a rapid onset of action in reducing migraine days and headache days at least moderate severity in EM patients and CM patients, respectively. Treatment effects were noted as early as the first day of injection in EM patients treated with fremanezumab quarterly and as early as the day after first injection in other patients. These results in Japanese and Korean patients align with those noted in subanalyses of fremanezumab and other anti-CGRP pathway monoclonal antibodies. In addition to the long-term efficacy and favorable safety and tolerability profile, the lack of need for titration, and flexible monthly or quarterly dosing schedule, the early onset of fremanezumab provides the potential to reduce barriers to adherence.

## Data Availability

The datasets analyzed during the current study are not publicly available but anonymized individual participant data are available from the corresponding author on reasonable request to achieve aims pre-specified in a methodologically sound research proposal.

## Abbreviations

ANCOVA: Analysis of covariance
CGRP: Calcitonin gene-related peptide
CI: Confidence interval
CM: Chronic migraine
EM: Episodic migraine
LSM: Least-squares mean
MMD: Monthly migraine days
MMRM: Mixed-effects model for repeated measures
SD: Standard deviation
SE: Standard error
SEM: Standard error of the mean
WMD: Weekly migraine days
